# Knowledge, Attitude, and Practice of Qualitative Research Among Health Sciences Faculty

**DOI:** 10.7759/cureus.44041

**Published:** 2023-08-24

**Authors:** Mohamud Mohamud, Alwaleed A Albarkani, Emad Masuadi, Abdullaziz A Alsahly, Abdulaziz I Alkudairy, Yazeed F Shalabi, Abdulrahman Faqih, Khaled A Alaukili, Saad J Alsahli

**Affiliations:** 1 Epidemiology, Somali Centres for Public Health, London, GBR; 2 Medical School, King Saud Bin Abdulaziz University for Health Sciences, Riyadh, SAU; 3 Research Unit, King Saud Bin Abdulaziz University for Health Sciences, Riyadh, SAU; 4 Biostatistics, King Abdullah International Medical Research Center, Riyadh, SAU; 5 Medical School, Alfaisal University College of Medicine, Riyadh, SAU; 6 Medical School, King Saud University, Riyadh, SAU

**Keywords:** ksau, qualitative methodologies importance, qualitative methodologies attitude, qualitative methodologies knowledge, qualitative methodologies practice, health sciences faculty, qualitative research

## Abstract

Background

The importance of qualitative research in health sciences is rising. Qualitative research needs more attention from healthcare practitioners. Hence, some questions in the healthcare field may only be answered through qualitative research methodologies. In this study, we aimed to assess the knowledge, practice, and attitude among health sciences faculty about qualitative research.

Methodology

This cross-sectional study was conducted at King Saud Bin Abdulaziz University for Health Sciences (KSAU-HS). A convenient sampling technique was used to collect data from health sciences faculty participants. Participants were included from five different colleges, i.e., College of Medicine, Applied Health Sciences, Nursing, Pharmacy, and College of Dentistry. A 20-question, self-made questionnaire was given to each participant. The questionnaire had five attitude questions, 10 knowledge questions, and five practice questions.

Results

A total of 236 participants completed the study questionnaire. The majority of the study participants (198, 84%) had an overall poor knowledge of qualitative research methodologies. Most participants (214, 91%) agreed that qualitative research is important in health sciences. More than half of the participants had never attended a qualitative methods workshop (140, 59%). About three-quarters of the participants (175, 74%) had never participated in a qualitative research project.

Conclusions

The overall knowledge and practice of qualitative research methodologies were poor among KSAU-HS health sciences faculty while they had a good attitude toward its importance in health sciences.

## Introduction

The importance of qualitative research is rising in healthcare [1,2]. Hence, many quantitative methodology publications do not provide a sufficient answer to the questions asked [3]. Although the use of qualitative research methodologies has increased, there is poor awareness in the broader field [1,4]. The rare practice of qualitative research methodologies in healthcare research has led to a lack of understanding of its methods in healthcare [2]. Qualitative research has several applications in the medical field, and selecting the appropriate qualitative research methodology to answer a specific question is important [5].

Management of chronic symptoms or diseases can be complex in adolescence and qualitative methods can make it easier to approach and understand [3]. Qualitative research can generate insights into patients’ experiences, beliefs, and attitudes, which patients may not always express in clinical settings [6]. Qualitative research addresses why and how research questions and provides a deeper understanding of context [7,8]. Moreover, it makes it possible to ask questions that cannot be answered easily with numbers [7,8]. Finally, qualitative research makes an important contribution to evidence-based practice in healthcare [9].

This study aimed to assess the knowledge, attitude, and practice toward qualitative research in health sciences faculty. The research intended to identify the different factors that led to higher knowledge levels of qualitative research. Moreover, this study aimed to raise awareness of qualitative research among health sciences faculty at King Saud Bin Abdulaziz University for Health Sciences (KSAU-HS).

## Materials and methods

Study design and area settings

A cross-sectional study was conducted at KSAU-HS in Riyadh province, Saudi Arabia.

Identification of study participants

The study population consisted of all faculty members from the College of Medicine, Pharmacy, Applied Health Sciences, Dentistry, and College of Nursing at KSAU-HS. English language teachers and teaching assistants were excluded from the study. The population size was 1,441. A sample size of 228 was recommended to achieve a margin of error of 5% or less, a confidence level of 90%, and a response distribution of 50%.

Data collection process

Data were collected manually by distributing a self-administered questionnaire. The questionnaire comprised a consent form, a demographic profile, five attitude questions, 10 knowledge questions, and five practice questions. The questionnaire was only available in the English language. It was self-developed and not validated before use.

A convenience sampling technique was used to collect data from faculty members at a university. The research team was divided into three data collection groups, and each group was assigned several colleges to survey. Data collectors visited faculty offices at different times to identify available faculty and obtain their consent to participate in the study. As faculty offices were located both on campus and at the hospital, data collectors also visited hospital department offices to identify potential participants. Duplicate questionnaire completion was avoided by asking faculty if they had previously completed the study.

Data analysis

The data were collected, reviewed, and then fed to SPSS Statistics for Windows version 21.0 (IBM Corp., Armonk, NY, USA). All statistical methods used were two-tailed, with an alpha level of 0.05 and p-values less than or equal to 0.05 denoting significance.

Overall knowledge level regarding qualitative research was assessed by summing up discrete scores for different correct awareness items. The overall knowledge score was categorized as poor if the participant score was less than 60% of the overall score and as good if the participant score was 60% or more of the overall score.

Descriptive analysis was done by prescribing frequency distribution and percentage for study variables including participants’ personal data, college, and years of teaching experience. Knowledge questions were tabulated with their scores, while overall knowledge was graphed. Cross-tabulation was used to show participants’ knowledge of qualitative research associated with their practice and was carried out using Pearson’s chi-square test for significance and exact probability test if there were small frequency distributions.

## Results

A total of 236 participants completed the study questionnaire. The majority of the participants were from the College of Medicine (96, 41%), followed by the College of Applied Health Sciences (58, 25%), College of Nursing (41, 17%), College of Pharmacy (26, 11%), and College of Dentistry (15, 6%). A total of 158 (67%) participants were males. Regarding years of teaching experience, the majority of the participants had two to five years of teaching experience (84, 36%). Moreover, 77 (33%) participants had a master’s degree (Table 1).

**Table 1:** Personal characteristics of study participants.

Personal data	No	%
Age in years		
25–35	83	35.2%
36–45	104	44.1%
>45	49	20.8%
Gender		
Male	158	66.9%
Female	78	33.1%
College		
Medicine	96	40.7%
Applied health sciences	58	24.6%
Nursing	41	17.4%
Pharmacy	26	11.0%
Dentistry	15	6.4%
Years of teaching experience		
<2 years	40	16.9%
2–5	84	35.6%
6–10	73	30.9%
11+	39	16.5%
Highest degree attained		
Bachelors	66	28.0%
Masters	77	32.6%
PhD	48	20.3%
MD	45	19.1%

Table 2 provides the knowledge details of the health sciences faculty at KSAU-HS about qualitative research for each question answered. The choices that were given for each question can be found in the Appendices. Most study participants (198, 84%) had an overall poor knowledge. Only 38 (16%) participants had a good knowledge level. The overall knowledge score was categorized as poor if the participant score was less than 60% of the overall score and good if the participant score was 60% or more of the overall score.

**Table 2:** Knowledge of qualitative research among participants.

Knowledge items	Correct answer	
	N	%
Phenomenological study includes all the following except for (Manipulation)	77	32.6%
A qualitative research design that examines the lived experiences of individuals is (Phenomenology)	46	19.5%
A guiding principle in deciding sample size in qualitative research is (Data saturation)	56	23.7%
Which of the following is not a critiquing qualitative research standard (Chance for committing Type I error)	51	21.6%
A qualitative method that focuses on the description and interpretation of cultural behavior (Ethnography)	71	30.2%
Which data collection method would you use when you have no pre-determined list of questions? (Unstructured interviews)	50	21.2%
The main method of collecting data from purposefully selected individuals is (In-depth interviews)	67	28.4%
Audio recording and note-taking are some of the main data collection features of (Qualitative research)	57	24.2%
Which of the following data collection methods interviewees need to have something in common (Focus group interviews)	54	22.9%
The recommended size of a typical focus group discussion (6–10)	76	32.2%

Table 3 presents the attitude of the health sciences faculty at KSAU-HS toward qualitative research. Most participants (214, 91%) agreed with the importance of qualitative research in health sciences, and 164 (70%) found qualitative research studies interesting. On the other hand, 136 (58%) thought that understanding qualitative research is difficult, and 101 (43%) participants thought that qualitative research is not appropriate for undergraduate students.

**Table 3:** Attitude toward qualitative research among participants.

Attitude items	Agree		Not sure		Disagree	
	N	%	N	%	N	%
Qualitative research in health sciences is important	214	90.7%	17	7.2%	5	2.1%
Qualitative research is not beneficial in the healthcare setting	15	6.3%	23	9.7%	198	84%
I find qualitative research studies interesting	164	69.5%	47	19.9%	25	10.6%
Understanding qualitative research is difficult	136	57.6%	53	22.5%	47	19.9%
Qualitative research is not appropriate for undergraduate students	101	42.8%	75	31.8%	60	25.4%

Table 4 presents qualitative research practice experiences among the health sciences faculty at KSAU-HS. More than half (140, 59%) of the study participants had never attended qualitative methods workshops. Moreover, about three-quarters (175, 74%) of the participants had never participated in a qualitative research project.

**Table 4:** Practice experience of qualitative research among participants.

Practice items	N	%
Attended qualitative methods workshops?		
Never	140	59.3%
1–3 times	66	28.0%
4–6 times	18	7.6%
7–10 times	7	3.0%
>10 times	5	2.1%
Taught qualitative research methods?		
Never	185	78.4%
1–3 times	31	13.1%
4–6 times	8	3.4%
7–10 times	5	2.1%
>10 times	7	3.0%
Participated or participating in a qualitative research project?		
Never	175	74.2%
1–3 times	45	19.1%
4–6 times	10	4.2%
7–10 times	2	0.8%
>10 times	4	1.7%
Supervised a qualitative research project?		
Never	195	82.6%
1–3 times	31	13.1%
4–6 times	7	3.0%
7–10 times	2	0.8%
>10 times	1	0.4%
Used qualitative research software for data analysis?		
Never	192	81.4%
1–3 times	32	13.6%
4–6 times	7	3.0%
7–10 times	3	1.3%
>10 times	2	0.8%

Table 5 shows the distribution of participants’ knowledge of qualitative research through their personal data. Participants whose ages were over 45 had an overall higher knowledge level than participants whose ages were between 25 and 35. Moreover, 14 (36%) participants with 11 years and above of teaching experience had a good knowledge level compared to three (8%) participants with teaching experience of less than two years. Moreover, good knowledge was detected among 15 (31%) of participants with a PhD compared to three (5%) of those with a bachelor’s degree.

**Table 5:** Distribution of participants’ knowledge of qualitative research through their personal data. P: Pearson’s chi-square test; $: exact probability test; *: p < 0.05 (significant).

Factors	Overall knowledge level				P-value
	Good		Poor		
	N	%	N	%	
Age in years					0.001*
25–35	6	7.2%	77	92.8%	
36–45	10	9.6%	94	90.4%	
>45	22	44.9%	27	55.1%	
Gender					0.358
Male	23	14.6%	135	85.4%	
Female	15	19.2%	63	80.8%	
College					0.425^$^
Applied health sciences	12	20.7%	46	79.3%	
Dentistry	1	6.7%	14	93.3%	
Medicine	12	12.5%	84	87.5%	
Nursing	9	22.0%	32	78.0%	
Pharmacy	4	15.4%	22	84.6%	
Years of teaching experience					0.001*
<2 years	3	7.5%	37	92.5%	
2–5	6	7.1%	78	92.9%	
6–10	15	20.5%	58	79.5%	
11+	14	35.9%	25	64.1%	
Highest degree attained					0.001*
Bachelors	3	4.5%	63	95.5%	
Masters	18	23.4%	59	76.6%	
PhD	15	31.3%	33	68.8%	
MD	2	4.4%	43	95.6%	

Table 6 shows the distribution of participants’ practice for qualitative research by their knowledge level. The majority of the participants with good knowledge of qualitative research had attended qualitative methods workshops (35, 92%). Further, 24 (71%) participants with good knowledge participated in a qualitative research project. Moreover, 24 (63%) participants with good knowledge taught qualitative research methods.

**Table 6:** Distribution of participants’ practice for qualitative research by their knowledge level. P: exact probability test; p < 0.05 (significant).

Practice	Overall knowledge level				P-value
	Good		Poor		
	N	%	N	%	
Attended qualitative methods workshops?					0.001*
Yes	35	92.1%	61	30.8%	
No	3	7.9%	137	69.2%	
Taught qualitative research methods?					0.001*
Yes	24	63.2%	27	13.6%	
No	14	36.8%	171	86.4%	
Participated or participating in a qualitative research project?					0.001*
Yes	27	71.1%	34	17.2%	
No	11	28.9%	164	82.8%	
Supervised a qualitative research project?					0.001*
Yes	19	50.0%	22	11.1%	
No	19	50.0%	176	88.9%	
Used qualitative research software for data analysis?					0.001*
Yes	19	50.0%	25	12.6%	
No	19	50.0%	173	87.4%	

## Discussion

The results of the study showed that the majority of the participants had an overall poor knowledge of qualitative research. More than half of the participants had never attended qualitative methods workshops. Moreover, about three-quarters of the participants had never participated in a qualitative research project. Further, More than half of the participants found qualitative research difficult to understand. Regardless, the majority of the participants agreed with the importance of qualitative research in health sciences.

Faculty participants who had attended qualitative methodologies workshops or had participated in qualitative research projects had significantly higher knowledge of qualitative research. Moreover, older faculty participants and those with higher education levels had more knowledge of qualitative research. Additionally, faculty with more teaching experience had higher knowledge levels.

In a study where 42 medical professionals were interviewed on their perceptions of qualitative research methodologies, results showed that the majority of the participants had nil knowledge of qualitative research methods [10]. Similarly, our research results showed poor overall knowledge of qualitative research among most of the health sciences faculty in KSAU-HS. Moreover, the majority of the 42 interviewed medical professionals considered qualitative methods to lack academic structure [10]. Others thought that qualitative methods are less important or supplementary to quantitative methods [10]. In contrast, the majority of our study participants agreed with the importance of qualitative research in health sciences. However, more than half of our participants thought that qualitative research is difficult to understand.

Another study showed that only 329 (9%) of the published research articles between 1998 and 2008 in nine different health services and management research journals used qualitative methods [11]. In addition, a previous study on the same nine journals between 1995 and 1997 reported that 14% of the articles used qualitative methods [12]. Our research results showed poor involvement in qualitative research methodologies. Almost three-quarters of the participants had never participated in qualitative research projects. Moreover, of those who had participated in qualitative research projects, the majority had minimal participation of one to three times. This may predict that similar to the mentioned studies, only a few of the 7,166 published research articles by KSAU-HS as of 2023 used qualitative research methods.

The findings of our study may have implications to advance evidence-based practice among KSAU-HS health sciences faculty, highlighting the important role of qualitative research in healthcare [9,13]. Qualitative research can provide valuable insights into the experiences of patients and providers, which can be used to improve the quality of care [14]. Qualitative research has many uses in healthcare [15]. Achieving higher knowledge and practice levels of qualitative research among KSAU-HS health sciences faculty can lead to the effective use of qualitative research methods in their healthcare practice.

This study has several limitations. First, as the study was conducted at a single university, the results may not be generalizable to other settings. Second, the study used a convenience sampling method, which may have introduced observer bias into the results. Third, the questionnaire was not validated before its use, which may have affected the accuracy of the results. Finally, the study did not assess the specific factors leading to poor participation in qualitative research methodologies.

## Conclusions

The purpose of this study was to assess the knowledge, attitude, and practice of qualitative research among KSAU-HS health sciences faculty. The findings of this study suggest that knowledge and practice of qualitative research are poor among the faculty, while the attitude toward the importance of qualitative research among health sciences faculty is good. Future research should address the limitations of this study and assess the specific factors that led to poor participation in qualitative research. This information can be used to further investigate possible interventions to raise knowledge and practice of qualitative research among KSAU-HS health sciences faculty.

## Appendices

**Table 7:** Section 1: Demographic profile.

Demographic	Response options				
Age groups	25–35	36–45		46 and above	
Gender	Male		Female		
University	KSAU-HS				
College	Medicine	Pharmacy	Applied Health Sciences	Dentistry	Nursing
Years of teaching experience	<2	2 to 5 years	6 to 10 years	11 years or more	
Highest degree attained	Bachelor	Masters	PhD	MD	

**Table 8:** Section 2: Attitude questions.

Item	Response options				
Qualitative research in health sciences is important	Strongly agree	Agree	Not sure	Disagree	Strongly disagree
Qualitative research is not beneficial in the healthcare setting	Strongly agree	Agree	Not sure	Disagree	Strongly disagree
I find qualitative research studies interesting	Strongly agree	Agree	Not sure	Disagree	Strongly disagree
Understanding qualitative research is difficult	Strongly agree	Agree	Not sure	Disagree	Strongly disagree
Qualitative research is not appropriate for undergraduate students	Strongly agree	Agree	Not sure	Disagree	Strongly disagree

Section 3: Knowledge questions

1. Phenomenological study includes all of the following except for:

a. Description

b. Analysis

c. Manipulation

d. Intuition

Correct answer: Manipulation

2. A qualitative research design that examines lived experiences of individuals is:

a. Mixed methods

b. Ethology

c. Typology

d. Phenomenology

Correct answer: Phenomenology

3. A guiding principle in deciding sample size in qualitative research is

a. Number of variables

b. Effect size

c. Data saturation

d. Sub-group analysis

Correct answer: Data saturation

4. Which of the following is not a critiquing qualitative research standard?

a. Heuristic relevance

b. Methodological congruence

c. Descriptive vividness

d. Chance for committing Type-I error

Correct answer: Chance for committing Type-I error

5. A qualitative method that focuses on description and interpretation of cultural behavior:

a. Symbolic interactionism

b. Ethnography

c. Grounded theory

d. Axial coding

Correct answer: Ethnography

6. Which data collection method would you use when you have no pre-determined list of questions?

a. Semi-structured interview

b. Unstructured interview

c. Multiple interview

d. Structured interview

Correct answer: Unstructured interview

7. The main method of collecting data from purposefully selected individuals is:

a. In-depth interview

b. Narration

c. Focus group

d. Structured group

Correct answer: In-depth interview

8. Audio recording and note-taking are some of the main data collection features of:

a. Exploratory design

b. Qualitative research

c. Questionnaire interviews

d. Categorical interview

Correct answer: Qualitative research

9. Which of the following data collection methods do interviewees need to have in common?

a. Exploratory interview

b. Focus group interview 

c. In-depth interview

d. Action research

Correct answer: Focus group interview

10. The recommended size of a typical focus group discussion is:

a. 10-15

b. 2-3

c. 6-10

d. 20 and above

Correct answer: 6-10

**Table 9:** Section 4: Practice questions.

Item	Response Options				
Attended qualitative methods workshops?	More than 10 times	7–10 times	4–6 times	1–3 times	Never
Taught qualitative research methods?	More than 10 times	7–10 times	4–6 times	1–3 times	Never
Participated or participating in a qualitative research project?	More than 10 times	7–10 times	4–6 times	1–3 times	Never
Supervised a qualitative research project?	More than 10 times	7–10 times	4–6 times	1–3 times	Never
Used qualitative research software for data analysis?	More than 10 times	7–10 times	4–6 times	1–3 times	Never
